# Clinical characteristics of central nervous system candidiasis due to *Candida albicans* in children: a single-center experience

**DOI:** 10.1186/s12879-022-07924-z

**Published:** 2022-12-16

**Authors:** Haijuan Xiao, Yiqing Miao, Linlin Liu, Wenya Feng, Shuping Liu, Lingyun Guo, Xin Guo, Tianming Chen, Bing Hu, Huili Hu, Fang Xu, Lianlian Han, Lili Ren, Wei Li, Gang Liu

**Affiliations:** 1 Department of Infectious Diseases, Beijing Children’s Hospital, Capital Medical University, Key Laboratory of Major Diseases in Children, Ministry of Education, National Center for Children’s Health, Beijing, 100045 China; 2 Research Unit of Critical Infection in Children, Chinese Academy of Medical Sciences, 2019RU016, Beijing, China; 3 Department of Respiratory Medicine, Children’s Hospital, Capital Institute of Pediatrics, Beijing, 100020 China; 4 Beijing Key Laboratory for Genetics of Birth Defects, Beijing Pediatric Research Institute, Key Laboratory of Major Diseases in Children, Ministry of Education, Genetics and Birth Defects Control Center, Beijing Children’s Hospital, Capital Medical University, National Center for Children’s Health, Beijing, 100045 China; 5 NHC Key Laboratory of System Biology of Pathogens, Institute of Pathogen Biology, Chinese Academy of Medical Sciences & Peking Union Medical College, Beijing, 100730 China

**Keywords:** Central nervous system candidiasis due to *Candida albicans* (CNSC), Invasive candidiasis (IC), Drug susceptibility test, Whole-exome sequencing (WES)

## Abstract

**Background:**

Central nervous system candidiasis due to *Candida albicans* (CNSC) in children is easily misdiagnosed and is associated with poor outcomes and a high mortality rate. There is no big data research or systematic review of CNSC.

**Methods:**

Patients diagnosed as CNSC with positive culture results of *Candida albicans* in Beijing Children’s Hospital affiliated to Capital Medical University from March 2010 to March 2019 were included. Patients receiving immunosuppressive therapy or transplantation, or with malignant tumours were excluded. We analysed the clinical characteristics, follow-up results, drug susceptibility tests and whole-exome sequencing (WES) results.

**Results:**

Thirty-three definitive patients were enrolled, including 22 males and 11 females. Twenty-five patients suffered from CNSC when they were less than 1 year old, and a total of 29 patients had high-risk factors. The main clinical manifestations were fever, convulsions, and positive neurological signs. Twenty-two patients had CNS infections alone, and 11 patients had CNS infections combined with invasive infections involving multiple sites. Twenty-seven cases had a positive CSF and/or blood culture at our hospital. All strains were susceptible to fluconazole, and 2 strains had intermediate susceptibility to voriconazole. As for amphotericin B, all the strains were wild type (WT). WES of 16 patients revealed 2 cases with *CARD9* mutations, who suffered from recurrent onychomycosis or thrush before.

**Conclusion:**

CNSC mostly existed in children younger than 1 year old, who all had underlying risk factors. CNSC patients with onset at an older age or with recurrent superficial fungal infections might have primary immunodeficiency.

**Supplementary Information:**

The online version contains supplementary material available at 10.1186/s12879-022-07924-z.

## Introduction

*Candida* is a genus of pathogenic fungi. They can cause candidemia by invading the bloodstream under specific circumstances or cause infections of certain deep organs (including the central nervous system (CNS), lung, digestive tract, urinary system, and bones and joints). These diseases are collectively referred to as invasive candidiasis (IC) [1]. IC ranks first in invasive fungal diseases (IFDs). *Candida albicans* is a well described fungal pathogen that could lead to IC in humans and generate high healthcare cost worldwide [2].

Central nervous system candidiasis due to *Candida albicans* (CNSC) rarely occurs in children, and it has occult onset, a prolonged disease course, and lacks specificity in clinical manifestations. The most important imaging manifestation of CNSC is meningoencephalitis [3]. Other manifestations include multiple brain abscesses with ring enhancement, intracranial vasculitis, hydrocephalus, and craniopathy [3]. CNSC may be accompanied by granuloma, haemorrhagic necrosis or demyelination in children [4]. Positive culture is still the gold standard for the diagnosis of fungal infection, however, fungal culture has a low overall positive rate (21–71%), takes a long time (2–5 days), and is affected by many factors, such as antifungal drugs, sample collection site and time [5]. The most frequently reported monogenic immunodeficiency disease that causes IC and CNSC is *Caspase Recruitment Domain Family Member 9* (*CARD9*) defects, followed by *Autoimmune Regulator* (AIRE), *Signal Transducer And Activator Of Transcription 1* (*STAT1*), *STAT3*, and *Interleukin 17F* (*IL17F*) defects [6–8].

CNSC in children is easily misdiagnosed. It is associated with poor outcomes and a high mortality rate. However, there is no big data research or systematic review of CNSC. This study summarized and analysed the clinical data, pathogen results and whole-exome sequencing (WES) results of CNSC in our hospital in the last 10 years, providing a reference for the timely detection, treatment, and prevention of CNSC.

## Methods

### Study subjects

The retrospective study was approved by the Ethics Committee of Beijing Children’s Hospital, Capital Medical University, and performed according to the Declaration of Helsinki. The written informed consents for participation in the study were obtained from the parents of participants. Patients aged 0–18 years who were diagnosed with CNSC and hospitalized between March 2010 and March 2019 at Beijing Children’s Hospital affiliated to Capital Medical University and who had complete medical records were included in this study. Suspected cases without positive pathogen findings were excluded. Immunosuppressive therapy, organ or bone marrow transplantation, and malignant tumours were also excluded. Personal information, past medical history, clinical manifestations, test results, imaging data, related complications, treatment regimens, post-discharge outcomes, pathogen detection and WES results were collected. The following information was obtained by telephone follow-up and outpatient visits: the full contents of the children’s version of the extended Glasgow Outcome Scale, medication and surgical treatment after discharge, and sequelae (e.g., symptomatic epilepsy, hearing impairment, mental retardation, delayed gross motor or fine motor development). Based on the prognosis at follow-up, the patients were divided into a deceased group and a survival group.

High-risk factors included extreme prematurity, extremely low birth weight, perinatal hypoxia, immunodeficiency, intensive care unit (ICU) admission, intubation history, broad-spectrum antibiotics, cerebrospinal fluid (CSF) leakage, the presence of prosthetic devices (including central venous catheters and ventricular-peritoneal shunts), history of surgery or trauma within the past 3 months, diabetes mellitus, total parenteral nutrition, hemodialysis therapy, and recurrent thrush or onychomycosis. Complications included infection-associated complications (septic shock, diffuse intravascular coagulation, bone marrow suppression, and multiple organ failure), neurological complications (e.g., seizures, neurodevelopmental sequelae, subdural effusion, ependymitis, hydrocephalus, brain abscess, cerebral infarction, cerebral haemorrhage, venous sinus thrombosis, and cerebral herniation), and treatment-related complications (e.g., adverse drug reactions, catheter-related complications).

### Clinical definitions

CNS infections: CNS infections include meningitis, encephalitis, and brain abscesses, and could be caused by pathogens (e.g., bacteria, virus, fungi, and tuberculosis). The diagnosis is typically made by identifying clinical symptoms and signs, evaluation of serology and CSF, demonstration of histopathology, and neuroimaging modalities [9, 10].

CNSC: Clinical diagnosis meets the criteria for CNS infections, and *Candida albicans* is cultured from sterile body fluids (e.g., blood, CSF, and intra-articular effusion) [11, 12].

IFD: Confirmed cases meet the requirement of a positive fungal culture of body fluids or tissues that are usually sterile (e.g., blood, CSF, lung tissue, and deep lymph nodes) and exhibit relevant symptoms and signs [13, 14].

IC: The diagnostic criteria are based on IFD. Notably, the aseptic sites referred to in the standard do not include all organs in contact with the external environment, such as the respiratory tract, genitourinary tract, and digestive tract, because these organs are common sites of *Candida* colonization [5].

Community-acquired infections: Onset occurs within 48 h of admission or more than 48 h after discharge.

Hospital-acquired infections: Onset occurs more than 48 h after admission or within 48 h after discharge in patients who have no infection symptoms and are not in a latent period at admission.

### Fungal culture and drug susceptibility test

Samples, such as blood, CSF, and intra-articular effusion, were inoculated onto Sabourand’s agar medium and incubated in an incubator at 37 °C for 48 h. Suspicious colonies on the plates were further separated and purified, and the species were identified by Matrix-Assisted Laser Desorption/Ionization Time of Flight Mass Spectrometry (MALDI-TOF MS) or Internal Transcribed Spacer (ITS) sequencing. The drug susceptibility tests were performed using an ATB Fungus 3 kit (Bio-Mérieux, France). The minimum inhibitory concentration (MIC) clinical breakpoints of fluconazole and voriconazole for *Candida albicans* were interpreted according to clinical and laboratory standards institute (CLSI) M60: fluconazole—susceptible (S) ≤ 2 µg/mL, susceptible-dose dependent (SDD) = 4 µg/mL, and resistant (R) ≥ 8 µg/mL; voriconazole—S ≤ 0.12 µg/mL, intermediate (I) = 0.25–0.5 µg/mL, and R ≥ 1 µg/mL [15]. There were no MIC clinical breakpoints for amphotericin B, and our study used epidemiological cutoff value (ECV, 2 µg/mL) to determine whether the strain was wide type (WT) or non-wild type (NWT) according to CLSI M59 [16]. There were no MIC clinical breakpoints of itraconazole and flucytosine for *Candida albicans* based on CLSI methods.

### WES

In attempt to identify an underlying etiological factor for susceptibility to CNSC, WES was done on a subset of patients with their parents’ approval. DNA was isolated from peripheral blood samples obtained from patients and parents using the Gentra Puregene Blood Kit (Qiagen, Hilden, Germany). Whole exome was captured using a SureSelect Human All Exon Kit (Agilent Technologies, Santa Clara, CA, USA). WES was performed on an Illumina Hiseq X Analyzer (Illumina, San Diego, CA) with 150 base paired-end runs. Sequence reads were mapped to the GRCh37/hg19 human reference genome. Variants were annotated and filtered by TGex (http://app.genecards.cn), and selection criteria referred to the principle described in the published paper [17]. Variants were classified according to the American College of Medical Genetics and Genomics and the Association for Molecular Pathology (ACMG/AMP) interpretation standards and guidelines [18].

## Results

### General data

Based on the inclusion and exclusion criteria, this study included 33 children with CNSC, including 22 males and 11 females (Table 1). The age of onset was from 0 to 14 years old, and 21 patients (63.6%) had an age of onset from 0 to 6 months old. The median duration for diagnosis (from onset of symptoms to positive culture) was 35 days, and the average length of hospital stay was 40 days. Four patients were misdiagnosed as tuberculosis infection and received antituberculosis treatment. A total of 14 patients were discharged from the hospital after their conditions improved, and their average length of hospital stay was 69 days. There were 19 patients admitted in the first 5 years of the study period, including 4 patients with invasive infection involving multiple sites, and 14 patients admitted in the latter 5 years, including 7 patients with invasive infection involving multiple sites (Additional file 1: Fig. S1).

**Table 1:** General data of 33 cases of CNSC

General data	Cases (n)/Median (M)	Percentage (%)/Quartile (Q1, Q3)
Male	22	66.7
Age of onset		
0–6 m	21	63.6
7–12 m	4	12.1
1–2 y	3	9.1
3–6 y	0	0
7–10 y	2	6.1
11–14 y	3	9.1
Rural cases	19	57.6
Onset in spring and summer	21	63.6
Course of disease before hospitalization (d)	30	22.5, 60
Time for diagnosis from onset of symptoms (d)	35	25, 68.5
Length of hospital stay (d)	40	19.5, 69
Discharge outcome		
Improvement	14	42.4
Automatic departure against doctors’ advice	19	57.6

*CNSC* central nervous system candidiasis due to *Candida albicans*; *d* day; *m* month; *y* year

### High-risk factors

**Fig. 1:**
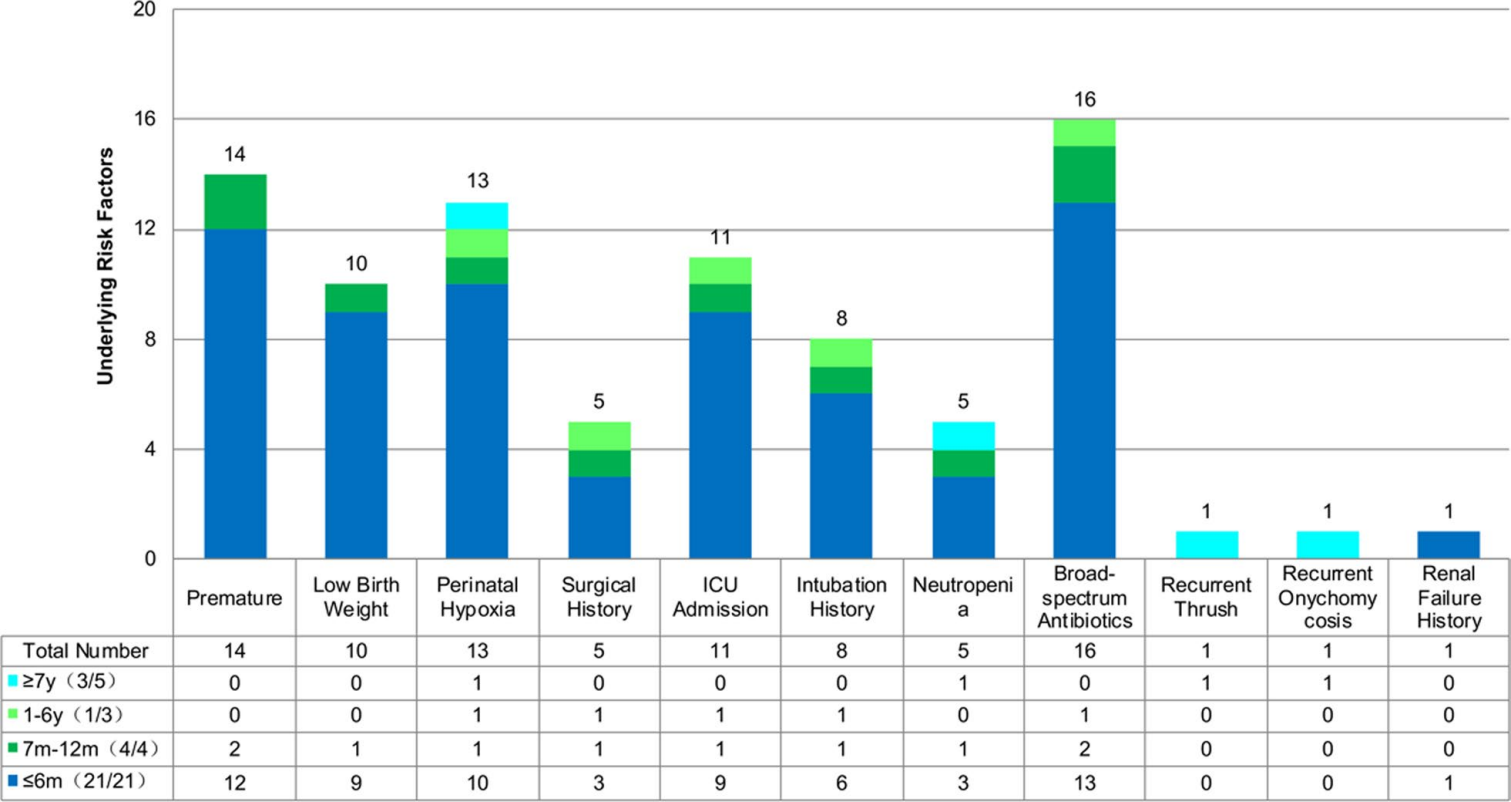
Underlying high-risk factors among different age groups. Twenty-nine patients had high-risk factors, which were present in all children younger than 1 year old. The proportion of patients with high-risk factors aged 1 year or older was 50%, significantly lower than the younger cases. Surgical history included 3 intracranial operations and 2 digestive tract operations

A total of 29 patients had high-risk factors, including preterm birth, low birth weight, perinatal asphyxia, surgery within 3 months before onset, admission to the ICU, tracheal intubation, application of broad-spectrum antibiotics, and neutropenia (Fig. 1). High-risk factors were present in all children younger than 1 year old, and the proportion (50%) of patients with high-risk factors was significantly lower in children aged 1 year or older. Four patients who had no clear disease history were all older children. Notably, among children aged 7 years or older, 1 had intrauterine hypoxia but normal growth and development, 1 had neutropenia that improved after treatment, 1 had a history of recurrent thrush, and 1 had a history of recurrent onychomycosis. The latter 2 patients did not receive regular treatment.

### Clinical characteristics

The main clinical manifestations of the patients included fever, convulsions, headache, vomiting, and enlarged head circumference. The main positive signs included changes in mental reactions (lethargy, irritability, and dysphoria), positive meningeal irritation signs, positive pathological signs, bulging fontanelle/high fontanelle tension, abnormal muscle strength/dystonia. Fever occurred in 75.7% of the patients, and the positivity rate for neurological examinations was 81.8% (Table 2).

**Table 2:** Clinical symptoms and signs of 33 cases of CNSC

Clinical symptoms and signs	Cases (n)	Percentage (%)
Fever	25	75.7
Convulsion	8	24.2
Headache	5	15.1
Vomiting	4	12.1
Cough	8	24.2
Diarrhea	2	6.0
Rash	3	9.0
Enlarged head circumference	5	15.1
Signs of nervous system	27	81.8
Changes in mental reactions^a^	19	57.5
Positive meningeal irritation signs	12	36.3
Positive pathological signs	7	21.2
Bulging fontanelle/high fontanelle tension	7	21.2
Sun set sign	6	18.1
Obtuse pupillary light reflex	3	9.0
Ocular motility disorder	2	6.0
Abnormal muscle strength/dystonia	5	15.1

*CNSC* central nervous system candidiasis due to *Candida albicans*

^a^Including lethargy, irritability, and dysphoria

### Laboratory tests

The white blood cell count was 10.32 × 10^9^/L, on average, and was < 4 × 10^9^/L in 2 patients. C-reactive protein (CRP) was normal in 87.8% of the patients. The CSF cell count was 292 × 10^6^/L, on average, and was > 1000 × 10^6^/L in 7 patients. The protein level in CSF significantly increased, while the glucose level decreased (Table 3). A total of 19 patients received β-D glucan assay (G test) of CSF and peripheral blood samples: 52% of the patients had positive G test of CSF. Ninety-seven percent of the patients had positive CSF cultures. One patient refused lumbar puncture due to past spinal surgery and was confirmed to have CNSC by clinical diagnosis and positive knee joint pus culture. The first cultures for twenty patients were positive and became negative after treatment. The time to negative culture conversion was 7–89 days, with an average of 15.5 (10.5, 22.5) days.

**Table 3:** Results of laboratory tests of CNSC patients

Laboratory tests	Cases (%)/M (Q1, Q3)
First peripheral blood cells (n = 33)	
WBC (× 10^9^/L)	10.3 (7.32, 12.65)
CRP < 8 mg/L	29 (87.8)
8 ≤ CRP ≤ 20 mg/L	2 (6.0)
CRP > 20 mg/L	2 (6.0)
Proportion of neutrophils (> 0.5)	14 (42.4)
First CSF (n = 32)	
WBC (× 10^6^/L)	292 (145, 910)
Proportion of multinuclear cells (> 0.5)	22 (68.7)
Protein (mg/L)	1732 (1209, 2929)
Glucose (mmol/L)	1.18 (0.74, 1.97)
G-test (n = 19)	
Positive in CSF and negative in blood	10 (52.6)
Both negative in CSF and in blood	9 (47.4)
Culture results (n = 33)	
Positive only in CSF	26 (78.8)
Positive in CSF and in blood	6 (18.2)
Positive in knee joint pus	1 (3.0)

*CNSC* central nervous system candidiasis due to *Candida albicans*; *CRP* C-reactive protein; *CSF* cerebrospinal fluid; *M* median; *Q* quartile; *WBC* white blood cell

### Imaging examination

**Fig. 2:**
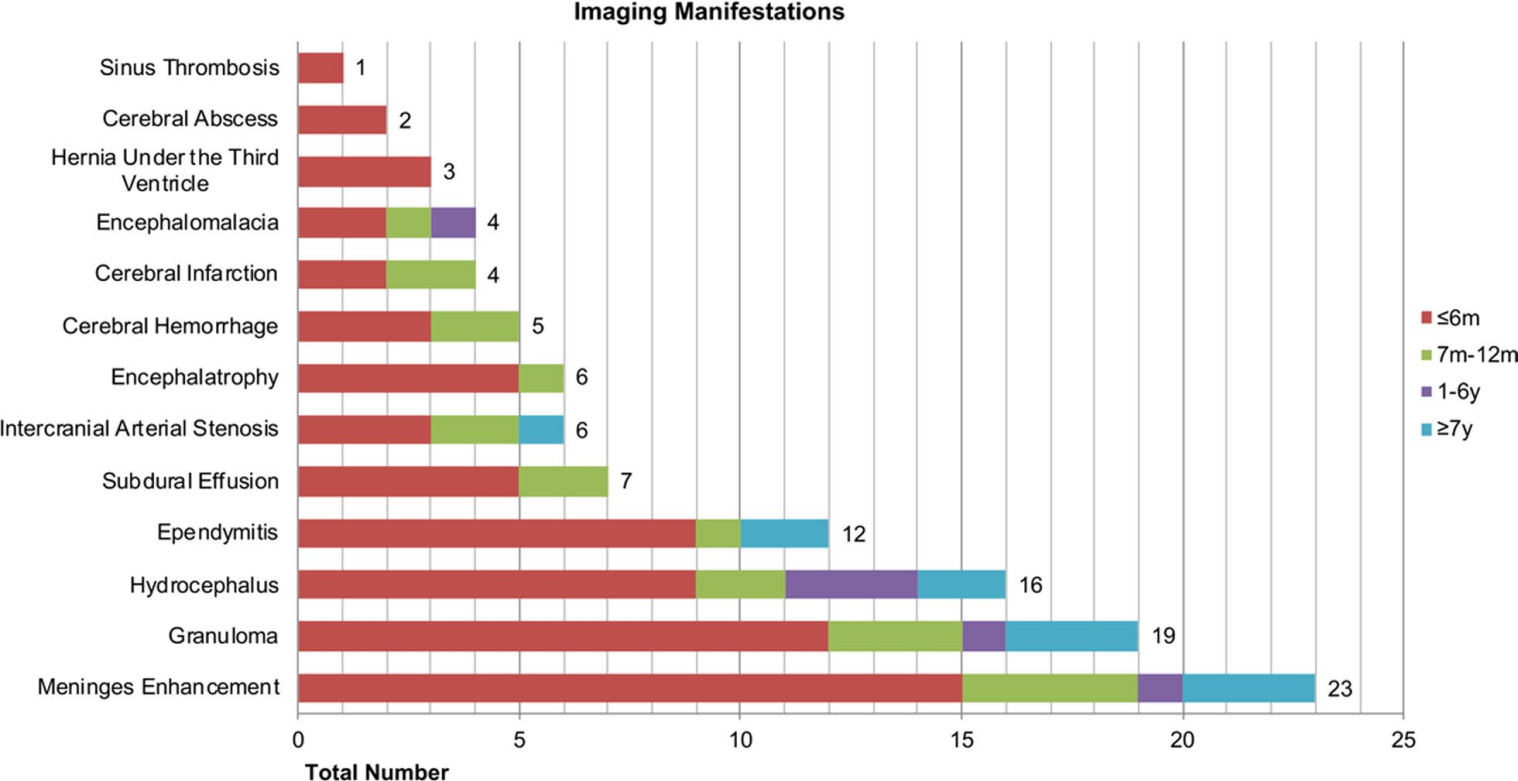
Imaging manifestations among different age groups of 32 patients. Meninges enhancement, granuloma, hydrocephalus, and ependymitis were the most common imaging features. Seven patients presented meningoencephalitis, 1 patient presented primary granuloma, and 3 patients presented vascular complications. The remaining 21 cases presented combinations of meningoencephalitis, brain abscess, granuloma, and vascular complications

**Fig. 3:**
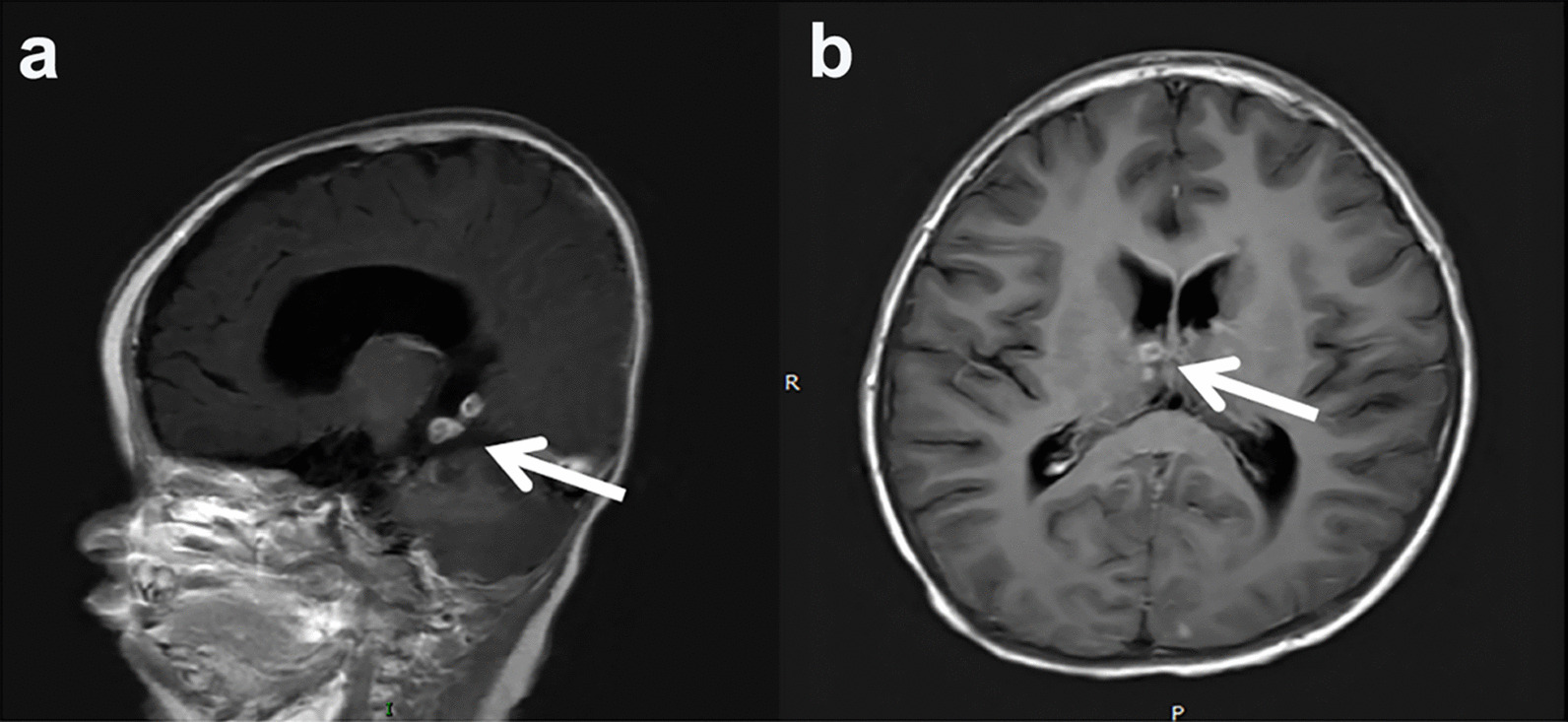
The typical granuloma changes of 2 CNSC cases according to enhanced brain MRI. **a** T1WI, sagittal scans; **b** T1WI, coronal scans. *CNSC* central nervous system candidiasis due to *Candida albicans*

**Fig. 4:**
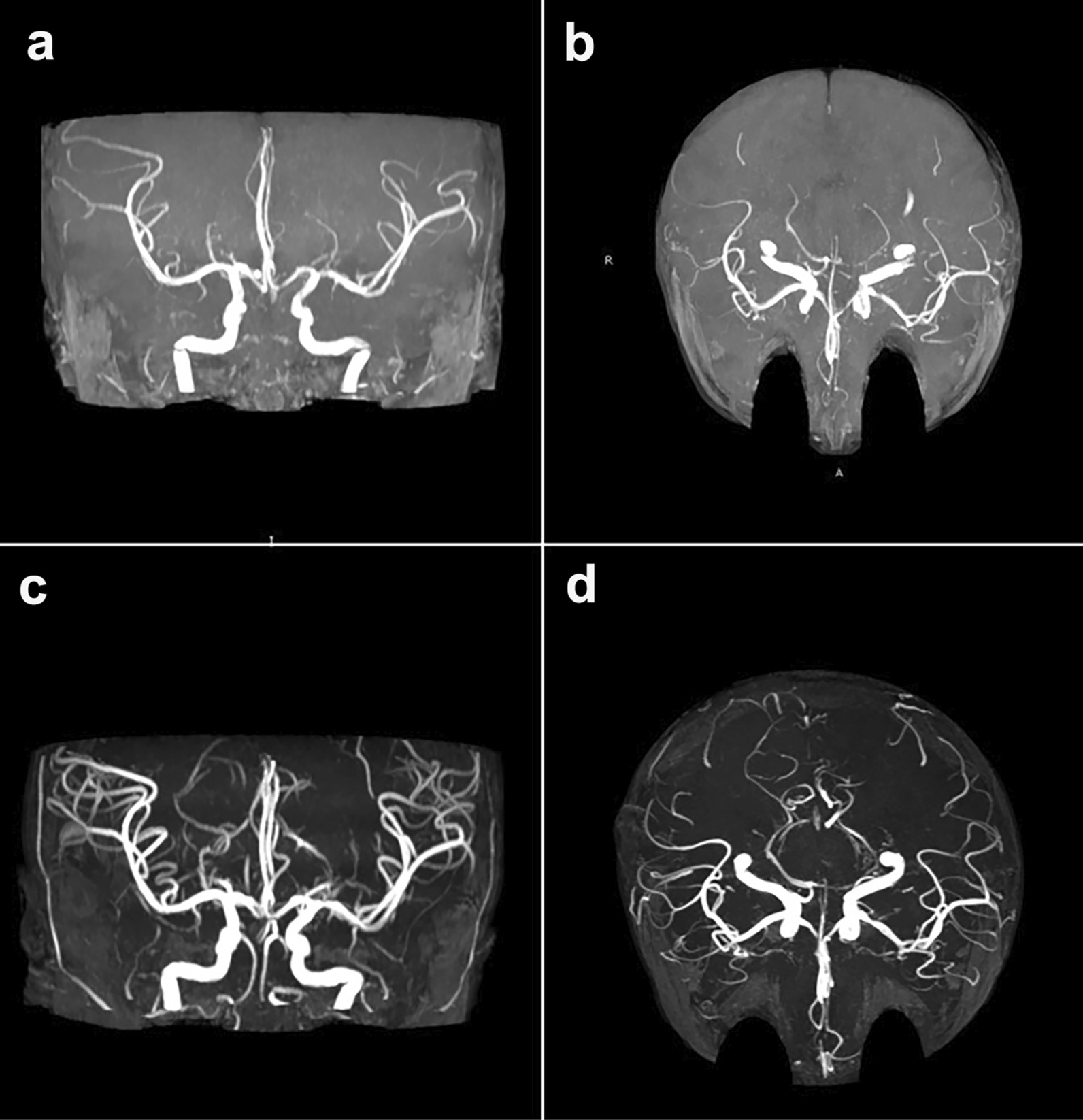
Intracranial vascular manifestations (artery stenosis) of 1 CNSC patient according to enhanced brain MRA. **a**, **b** before treatment; **c**, **d** after treatment. *CNSC* central nervous system candidiasis due to *Candida albicans*

In this study, intracranial CNSC lesions were classified into meningoencephalitis, brain abscess, primary granuloma, and vascular complication. Imaging examinations were performed for 32 patients, showing varying degrees of intracranial diseases, including meningeal enhancement, granuloma, hydrocephalus, ependymitis, subdural effusion, intracranial artery stenosis, brain atrophy, and cerebral haemorrhage (Fig. 2). Seven patients presented meningoencephalitis, 1 patient presented primary granuloma (Fig. 3), 3 patients younger than 6 months old presented vascular complications (Fig. 4), and the remaining 21 patients presented combinations of the above 4 types.

### Complications

No infection-related complications (e.g., septic shock) occurred in 33 patients during hospitalization. A total of 28 patients had neurological complications, including hydrocephalus (16), ependymitis (12), subdural effusion (7), cerebral haemorrhage (5), cerebral infarction (4), cerebral herniation in the lower third ventricle (3), cerebral venous sinus thrombosis (1), hearing impairment (4), symptomatic epilepsy (2), and optic nerve block (1). Fourteen children were treated with amphotericin B, including 2 with electrolyte imbalance and 2 with renal dysfunction. Five children were treated with amphotericin B liposomes, including 1 with electrolyte imbalance. Venous catheterization was performed in 13 patients. Five patients had catheter-related complications, including phlebitis (4) and venous thrombosis (2).

### Types of disease

In this study, there were 26 cases of community-acquired infection and 7 cases of hospital-acquired infection, and all children over 7 years old had community-acquired infections (Additional file 1: Table S1). Twenty-two patients had CNS infections alone, and 11 patients had CNS infections combined with invasive infections involving multiple sites (Additional file 1: Table S2, Fig. S1). In children with invasive infections involving multiple sites, CNS infections combined with bloodstream infection were most common (6). High-risk factors existed in all children with invasive infections involving multiple sites, who had a shorter average diagnosis time but a longer length of hospital stay (Table 4).

**Table 4:** Clinical features of patients with CNS infection alone and patients with invasive infections involving multiple sites

Clinical features	CNS infection alone (n = 22, cases (%)/M (Q1, Q3))	Invasive infections involving multiple sites (n = 11, cases (%)/M (Q1, Q3))
Male	14 (63.6)	8 (72.7)
Age of onset		
≤ 6 m	14 (63.6)	7 (63.6)
7–12 m	3 (13.6)	1 (9.0)
1–6 y	2 (9.0)	1 (9.0)
≥ 7 y	3 (13.6)	2 (18.1)
High risk factors	18 (81.8)	11 (100.0)
Premature birth	9 (40.9)	5 (45.4)
Low birth weight	6 (27.2)	4 (36.3)
Perinatal hypoxia	7 (31.8)	6 (54.5)
Surgical history	3 (13.6)	2 (18.1)
ICU admission	7 (31.8)	4 (36.3)
Intubation history	5 (22.7)	3 (27.2)
Neutropenia	4 (18.1)	1 (9.0)
Antibiotics	11 (50.0)	5 (45.4)
Length of hospital stay (d)	40 (27, 63)	41.5 (18, 72.75)
Time of diagnosis (d)	44 (10, 126)	36 (29.25, 75.75)

*CNS* central nervous system; *d* day; *M* median; *m* month; *Q* quantile; *y* year

### Prognostic analysis

**Fig. 5:**
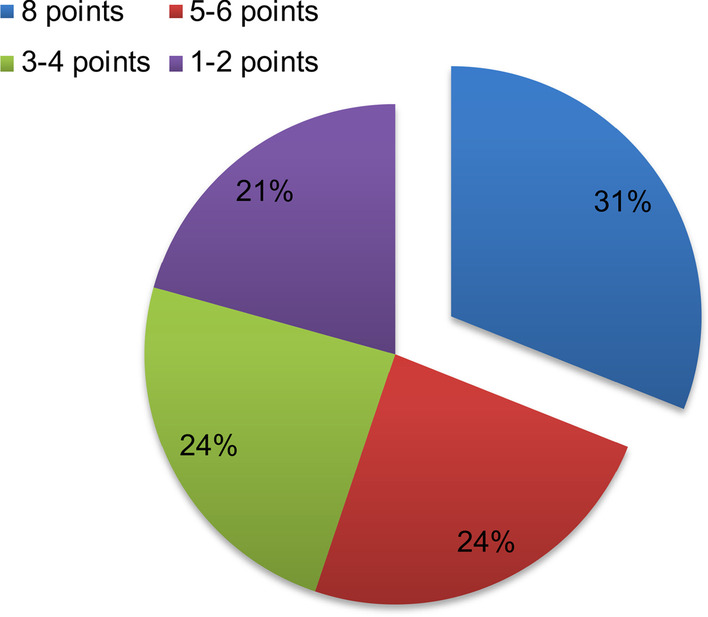
The prognosis scores during the follow-up of 29 patients according to the children’s version of the extended Glasgow Outcome Scale. Nine cases of death (8 points), 7 cases of severe disability, 7 cases of moderate disability, and 6 cases of good recovery were shown

During the follow-up, 4 patients were lost to follow-up, who were not included in the outcome analysis. Ultimately, 29 patients were followed up. The shortest follow-up time was 1 year, and the longest was 9 years. During follow-up, the children’s version of the extended Glasgow Outcome Scale was used for scoring prognosis. The results showed 9 cases of death (8 points), 7 cases of severe disability (5–6 points), 7 cases of moderate disability (3–4 points), and 6 cases of good recovery (1–2 points). The mortality rate for children with CNSC was 31.0%, and the disability rate was 48.2% (Fig. 5).

### Drug susceptibility test results and antifungal drugs

In this study, 27 patients with positive CSF and/or peripheral blood fungal cultures had drug susceptibility test results. All strains were susceptible to fluconazole, and 2 strains had intermediate susceptibility to voriconazole (their voriconazole MICs were both 0.25 µg/mL, and fluconazole MICs were both 2 µg/mL). The amphotericin B MIC of 26 strains was ≤ 0.5 µg/mL, and only 1 strain was 2 µg/mL (Additional file 1: Table S3).

All patients received initial treatment after a confirmed positive fungal culture: 2 received triad treatment with fluconazole, flucytosine, and amphotericin B, 5 received flucytosine combined with amphotericin B, and 26 received fluconazole (Additional file 1: Table S4). Among the patients who received fluconazole as the initial treatment, 7 patients were treated with fluconazole alone for the whole disease course, and the remaining patients later received voriconazole, flucytosine combined with amphotericin B, or fluconazole combined with flucytosine and/or amphotericin B based on their conditions and treatment efficacy. Nineteen patients received intravenous antifungal therapy for 4 weeks, and 14 patients received intravenous antifungal therapy for less than 4 weeks. Other medical treatment regimens included intravenous immunoglobulin (IVIG), glucocorticoid, interferon, and granulocyte colony-stimulating factor (G-CSF) (Additional file 1: Table S5). In addition, due to the high proportion of children with hydrocephalus or subdural effusion, 15 patients received surgical treatment, including Ommaya reservoir implantation, third ventriculostomy, midbrain aqueduct fistulation, ventricular-abdominal shunt, lateral ventricular drainage.

### WES results

WES was conducted for 16 children. Two patients had *CARD9* mutations. One patient was a 10-year-old boy, with the following major manifestations: bone destruction, intermittent fever, irritability, blurred vision and history of recurrent onychomycosis. His knee joint pus culture was positive for *Candida albicans*. His head imaging manifestations were meningoencephalitis and vasculitis. The patient was diagnosed with IC (CNS, spine, right knee joint). Although his condition improved after 48 days of hospitalization, he continued to suffer from lameness, which had not improved at 2 years of follow-up. The gene detection indicated *CARD9* compound heterozygous mutations (NM_052813, c.246C>A, p.S82R, from his father, and c.1497delT, p.F499Lfs*? from his mother, which are mutation sites that have never been reported before) (Additional file 1: Fig. S2). The other child was a 13-year-old boy who mainly presented intermittent fever accompanied by coughing. He had a history of recurrent thrush. His CSF culture was positive for *Candida albicans*. His imaging manifestations were meningoencephalitis. The patient was diagnosed with CNSC, which improved after 45 days of hospitalization. The patient had no sequelae at 4 years of follow-up. Gene detection revealed 2 *CARD9* mutation sites (NM_052813, c.191_192insTGCT, p.L65Afs*58, het, and c.820dupG, p.D274Gfs*60, het; pathogenic mutations on both sites have been reported) [19]. However, his parents refused Sanger sequencing validation.

## Discussion

The existing literature aimed specially at CNSC in children was quite limited. The main focus of this study was children diagnosed with CNSC in our hospital, which promoted the understanding the clinical features, drug resistance characteristics, and underlying gene deficiency of this disease.

In our patients, up to 87.8% had high-risk factors, a value that is basically consistent with the results of previous studies [20, 21]. This study did not include CNSC patients with haematologic or solid tumours, bone marrow or organ transplantation, and immunosuppressive therapy. Moreover, CNSC patients in the ICU of our hospital were relatively rare, perhaps because of no positive CSF culture due to the active prophylactic application of antifungal therapy in these patients. In this study, 2 patients had *CARD9* mutations (for one of these patients, Sanger sequencing and pedigree validation were needed to verify the pathogenicity of *CARD9* mutations), further confirming that *CARD9* defects are closely related to CNSC.

The clinical manifestations and CSF indexes of patients with CNSC are similar to those of purulent meningitis; therefore, the differentiation between CNSC and purulent meningitis is difficult in clinical practice and mainly relies on imaging findings, G tests and culture results. In this study, the imaging manifestations of patients were diverse. The most common manifestations were meningoencephalitis, and some manifestations were combined with cerebrovascular complications. The high proportion of hydrocephalus was perhaps related to the significantly elevated protein levels in CSF. A meta-analysis showed that the diagnostic sensitivity and specificity of serum G test for patients with IFD were 79.1% and 87.7%, respectively [22]. Another study showed that CSF G test had a sensitivity of up to 100% and a specificity as high as 95–98% in patients with non-*Candida* CNS fungal infections [23, 24]. Currently, the positive threshold value for CSF G test in children has not been established internationally. The positive threshold value for serum G tests was applied (i.e., > 60 ng/mL was considered positive) in this study, and the positivity rate (i.e., sensitivity) for CSF G tests was 52.6%, which is lower than the results of the above studies perhaps because of the small total sample size, different sample types or pathogens, and different medication histories.

In the 2016 Clinical Practice Guideline, the Infectious Diseases Society of America (IDSA) recommend the use of amphotericin B in neonates with CNSC and the use of flucytosine when the efficacy of amphotericin B is poor, the use of fluconazole for step-down therapy in patients who respond well to initial treatment, the use of amphotericin B liposomes and echinocandins with caution, and the removal of catheters that may lead to retrograde infection, such as central venous catheters. CNSC treatment should continue until symptoms, signs, and abnormal imaging findings resolve. The treatment for candidemia should continue until 2 weeks after a negative blood culture [25]. In this study, the major treatment regimen was amphotericin B combined with flucytosine, a finding that was consistent with the guideline recommendations. Some patients also received fluconazole for triad treatment. When candidemia is suspected but the culture results are negative and the pathogen cannot be confirmed, fluconazole and echinocandins are recommended as the initial empirical therapy in many guidelines, including the IDSA guidelines [24]. However, because the concentrations of echinocandins are low in the CNS and urinary tract, their use in CNSC treatment is limited. In this study, most patients received fluconazole as the initial treatment.

This was a single-centre retrospective study, only including confirmed CNSC patients but not including clinically diagnosed or suspected cases. Therefore, the results might be biased. Due to the limited sample size and different ages of our patients, death risk factors were not analysed. In addition, only 27 patients had clear drug susceptibility test results, and only 16 patients underwent WES. The limited data made it impossible to investigate in depth the relationship between fungal drug resistance and CNSC and the relationship between genetic susceptibility and CNSC. In the future, it is necessary to expand the sample size and use prospective studies to further analyse the death risk factors and fungal drug resistance in CNSC and to explore the best treatment regimen.

## Conclusion

CNSC in children occurred mostly in those younger than 1 year old. CNSC children with disease onset within 1 year old and with invasive infections involving multiple sites both had high-risk factors. No drug-resistant or NWT strains were found in the pathogenic strains, and the mortality rate and disability rate of CNSC were high. Two CNSC patients had *CARD9* mutations, so genetic testing should be further performed in patients with onset at an older age and no underlying disease or with recurrent superficial fungal infections.

## Supplementary Information

**Additional file 1: Table S1.** Types of infection of 33 CNSC patients. **Table S2.** Sites of infection of 33 CNSC patients. **Table S3.** Drug susceptibility test results of 27 strains of *Candida albicans*. **Table S4.** Antifungal drugs used in CNSC patients. **Table S5.** Other medical treatment regimens used in CNSC patients. **Figure S1.** Number of patients with CNSC and patients with different sites of infection from March 2010 to March 2019. **Figure S2.** The WES result of 1 CNSC patient indicated *CARD9* compound heterozygous mutations.

## Data Availability

All data generated or analyzed during this study is included in this published article and its Additional file 1.

## Abbreviations

ACMG/AMP: American College of Medical Genetics and Genomics and the Association for Molecular Pathology
*AIRE*: *Autoimmune Regulator*
*CARD9*: *Caspase Recruitment Domain Family Member 9*
CLSI: Clinical and Laboratory Standards Institute
CNS: Central nervous system
CNSC: Central nervous system candidiasis due to *Candida albicans*
CRP: C-reactive protein
CSF: Cerebrospinal fluid
ECV: Epidemiological cutoff value
G-CSF: Granulocyte colony-stimulating factor
IC: Invasive candidiasis
ICU: Intensive care unit
IDSA: Infectious Diseases Society of America
IFDs: Invasive fungal diseases
*IL17F*: *Interleukin 17F*
ITS: Internal Transcribed Spacer
IVIG: Intravenous immunoglobulin
MALDI–TOF MS: Matrix-Assisted Laser Desorption/Ionization Time of Flight Mass Spectrometry
MIC: Minimum inhibitory concentration
NWT: Non-wild type
SDD: Susceptible-dose dependent
*STAT1*: *Signal Transducer And Activator Of Transcription 1*
WES: Whole-exome sequencing
WT: Wild type
