# Supplementary material for: Clinical characteristics of central nervous system candidiasis due to Candida albicans in children: a single-center experience
Source: BMC Infect Dis. 2022 Dec 16;22:945. doi: 10.1186/s12879-022-07924-z (PMC9756474; doi:10.1186/s12879-022-07924-z)
Supplement: Supplementary file 1 — Additional file 1: Table S1. Types of infection of 33 CNSC patients. Table S2. Sites of infection of 33 CNSC patients. Table S3. Drug susceptibility test results of 27 strains of Candida albicans. Table S4. Antifungal drugs used in CNSC patients. Table S5. Other medical treatment regimens used in CNSC patients. Figure S1. Number of patients with CNSC and patients with different sites of infection from March 2010 to March 2019. Figure S2. The WES result of 1 CNSC patient indicated CARD9 compound heterozygous mutations. [file 12879_2022_7924_MOESM1_ESM.pdf]

Additional file 1

Discussion

Low expression of granulocyte-macrophage colony-stimulating factor (GM-CSF)-related genes in peripheral blood mononuclear cells and decreased levels of secreted GM-CSF in peripheral blood were detected in 1 patient with CNSC and homozygous *CARD9* mutation [1]. Six days after treatment with GM-CSF, the clinical symptoms of the patient significantly improved, CSF culture turned to negative, and the number of cells and protein levels in CSF decreased. In this study, 2 patients with neutropenia during the disease course received granulocyte colony-stimulating factor (G-CSF) treatment. After neutrophils returned to normal levels, treatment was stopped. The condition of 1 patient significantly improved, and the other patient eventually recovered. However, there are few reports on the treatment of CNSCs with G-CSF and GM-CSF; therefore, further studies are needed to further clarify their therapeutic value.

A study on hospitalized children with IC found 44 fluconazole-resistant strains, 3 amphotericin B-resistant strains, and 2 echinocandin-resistant strains [2]. In this study, all the strains were fluconazole-susceptible, and only 2 strains had intermediate susceptibility to voriconazole. As for amphotericin B, all the strains were WT. The small sample size and the dominance of community-acquired infection in this study might have contributed to that result. A study in 2017 reported that the median time to negative conversion of blood culture and CSF culture for 120 children with IC was 5.08 days, while the median time to negative conversion of blood culture and CSF culture for 20 patients in our study was 15.5 days (range: 7-89 days) [3]. The longer median time to negative conversion in our study was perhaps because the type of cultured specimens was mainly CSF and the treatment of CNS infections was more difficult. In addition, our study might have deviations due to the small number of cases.

Previous studies on IFD mainly focus on populations with underlying diseases such as hematologic diseases and malignant tumors, patients admitted to ICUs, or neonatal populations and rarely reported other populations. They show that CNSC and/or candidemia are/is the leading causes/cause of mortality and neurological sequelae among children with IC and very low birth weight [4]. It has been reported that the overall mortality rate for patients with IC varies between 10% and 70% and reaches 90% if patients have CNS involvement [5]. The

results of this study showed that the overall mortality rate for children with CNSC but without the above underlying diseases was 31%, a finding similar to the mortality rate for children with concomitant IC and neutropenia (30%) [6], the mortality rate for children with IC in ICUs (29.6%) [7], and the mortality rate for neonates with concomitant IC and CNS involvement (35-40%) [8]. Compared with other CNS infections, the mortality rate for children with CNSC in this study was higher than the previously reported mortality rate for children with tuberculous meningitis (19%) [9] and the mortality rate for children with purulent meningitis (3%) [10]. The percentage of patients with different degrees of CNSC sequelae was 48% in the survival group, suggesting that clinicians needed to pay more attention to the possibility of CNSC development in children without serious underlying diseases, be more alert to this disease, and actively perform early diagnosis and adequate-dose, full-course antifungal therapy.

References

1. Gavino C, Cotter A, Lichtenstein D, et al. CARD9 deficiency and spontaneous central nervous system candidiasis: complete clinical remission with GM-CSF therapy. Clin Infect Dis. 2014;59(1):81-4.
2. Lai MY, Hsu JF, Chu SM, et al. Breakthrough candidemia in children: clinical and microbiological characteristics, therapeutic strategies and impact on outcomes. Future Microbiol. 2017;12:695-705.
3. Harrington R, Kindermann SL, Hou Q, Taylor RJ, Azie N, Horn DL. Candidemia and invasive candidiasis among hospitalized neonates and pediatric patients. Curr Med Res Opin. 2017;33(10):1803-12.
4. Adams-Chapman I, Bann CM, Das A, et al. Neurodevelopmental outcome of extremely low birth weight infants with Candida infection. J Pediatr. 2013;163(4):961-7 e3.
5. Goralska K, Blaszkowska J, Dzikowiec M. Neuroinfections caused by fungi. Infection. 2018;46(4):443-59.
6. Fortun J, Gioia F. Invasive candidiasis in the neutropenic patient. Rev Esp Quimioter. 2017;30(suppl 1):22-5.
7. Gong X, Luan T, Wu X, et al. Invasive candidiasis in intensive care units in China: Risk factors and prognoses of Candida albicans and non-albicans Candida infections. Am J Infect Control. 2016;44(5):e59-63.
8. Drummond RA, Lionakis MS. Candidiasis of the Central Nervous System in Neonates and Children with Primary Immunodeficiencies. Curr Fungal Infect Rep. 2018;12(2):92-7.
9. Mezochow A, Thakur K, Vinnard C. Tuberculous Meningitis in Children and Adults: New Insights for an Ancient Foe. Curr Neurol Neurosci Rep. 2017;17(11):85.
10. Collaborators GBDM. Global, regional, and national burden of meningitis, 1990-2016: a systematic analysis for the Global Burden of Disease Study 2016. Lancet Neurol. 2018;17(12):1061-82.

Table S1 Types of infection of 33 CNSC patients.

Types of infection	≤6m (n=21)	7m-12m (n=4)	1y-6y (n=3)	≥7y (n=5)
Community-acquired infection	16 (76.2)	3 (75.0)	2 (66.7)	5 (100.0)
Hospital-acquired infection	5 (23.8)	1 (25.0)	1 (33.3)	0 (0.0)

CNSC, central nervous system candidiasis due to *Candida albicans*.

Table S2 Sites of infection of 33 CNSC patients.

Sites of infection	Cases (n)	Percentage (%)
CNS infection alone	22	66.7
CNS infection combined with		
Bloodstream infection	5	15.2
Pulmonary infection	1	3.0
Abdominal infection	1	3.0
Bone destruction	1	3.0
Multiple sites involved (≥3)		
CNS + bloodstream + urinary system	1	3.0
CNS + lung + abdomen	1	3.0
CNS + joint + bone	1	3.0

CNS, central nervous system; CNSC, central nervous system candidiasis due to *Candida albicans*.

Table S3 Drug susceptibility test results of 27 strains of *Candida albicans*.

Drugs	Strain	Susceptible/WT strain (%)	Intermediate strain (%)	Resistant/NWT strain (%)
Amphotericin B	27	27 (100) [a]	0	0
Fluconazole	27	27 (100)	0	0
Voriconazole	27	25 (92.5)	2 (7.4) [b]	0

[a] The amphotericin B MIC of 1 strain was 2μg/mL. [b] The voriconazole MICs of 2 strains were both 0.25μg/mL, and their fluconazole MICs were both 2μg/mL. MIC, minimum inhibitory concentration; WT, wild type; NWT, non-wild type.

Table S4 Antifungal drugs used in CNSC patients.

Initial treatment	Adjustment therapy	Cases (%)
Fluconazole	none	7 (21.2)
	Later changed for voriconazole	4 (12.1)
	Combined with flucytosine	2 (6.0)
	Combined with amphotericin B	1 (3.0)
	Combined with flucytosine and amphotericin B	7 (21.2)
	Later changed for flucytosine combined with amphotericin B	5 (15.1)
Flucytosine combined with amphotericin B		5 (15.1)
Triad treatment with fluconazole, flucytosine, and amphotericin B		2 (6.0)

CNSC, central nervous system candidiasis due to *Candida albicans*.

Table S5 Other medical treatment regimens used in CNSC patients.

Therapies	Cases (n)	Percentage (%)
IVIG	11	33.3
Glucocorticoid	8	24.2
Interferon	2	6.0
G-CSF	2	6.0
Surgical treatment	15	45.4

CNSC, central nervous system candidiasis due to *Candida albicans*; G-CSF, granulocyte-stimulating factor; IVIG, intravenous immunoglobulin.

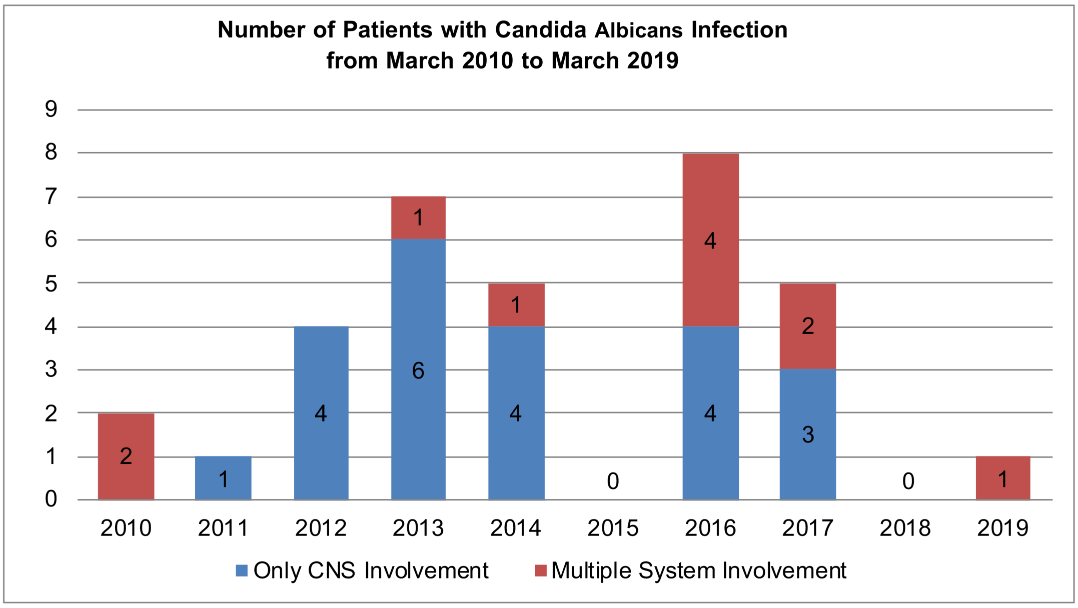

Fig. S1 Number of patients with CNSC and patients with different sites of infection from March 2010 to March 2019. Twenty-two patients suffered from CNS infection alone, and 11 patients had CNS infection combined with invasive infections involving multiple sites. CNS, central nervous system; CNSC, central nervous system candidiasis due to *Candida albicans*.

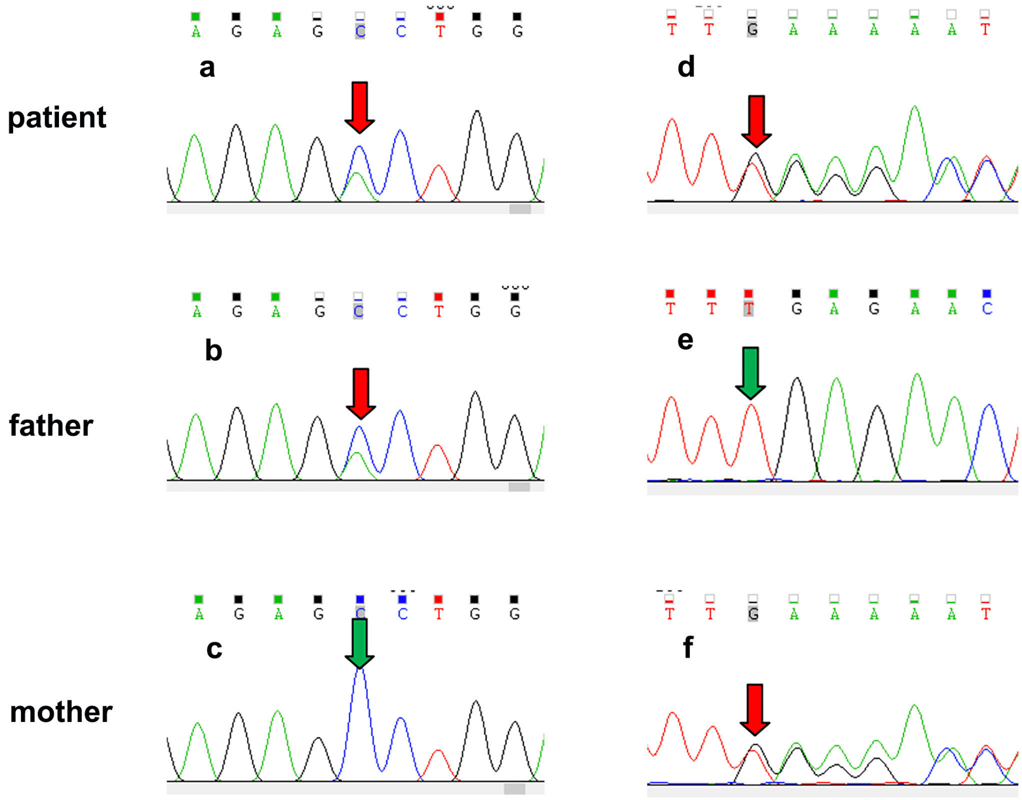

Fig. S2 The WES result of 1 CNSC patient indicated *CARD9* compound heterozygous mutations. **a-c**, NM_052813, c.246C>A, from his father; **d-f**, c.1497delT, from his mother. CNSC, central nervous system candidiasis due to *Candida albicans*; WES, whole-exome sequencing.
